# High intensity exercise during breast cancer chemotherapy - effects on long-term myocardial damage and physical capacity - data from the OptiTrain RCT

**DOI:** 10.1186/s40959-021-00091-1

**Published:** 2021-02-15

**Authors:** Josefin Ansund, Sara Mijwel, Kate A. Bolam, Renske Altena, Yvonne Wengström, Eric Rullman, Helene Rundqvist

**Affiliations:** 1 Department of Laboratory Medicine, Karolinska Institutet, Stockholm, Sweden; 2 Unit of Clinical Physiology, Karolinska University Hospital, Stockholm, Sweden; 3 Department of Neurobiology, Care Sciences and Society, Karolinska Institutet, Stockholm, Sweden; 4 Cancer Theme, Karolinska University Hospital, Stockholm, Sweden; 5 Department of Oncology and Pathology, Karolinska Institutet, Stockholm, Sweden

**Keywords:** Exercise oncology, Cardiotoxicity, Biomarkers

## Abstract

**Background:**

Adjuvant systemic breast cancer treatment improves disease specific outcomes, but also presents with cardiac toxicity. In this post-hoc exploratory analysis of the OptiTrain trial, the effects of exercise on cardiotoxicity were monitored by assessing fitness and biomarkers over the intervention and into survivorship.

Methods; Women starting chemotherapy were randomized to 16-weeks of resistance and high-intensity interval training (RT-HIIT), moderate-intensity aerobic and high-intensity interval training (AT–HIIT), or usual care (UC). Outcome measures included plasma troponin-T (cTnT), Nt-pro-BNP and peak oxygen uptake (VO_2peak_), assessed at baseline, post-intervention, and at 1- and 2-years.

**Results:**

For this per-protocol analysis, 88 women met criteria for inclusion. Plasma cTnT increased in all groups post-intervention. At the 1-year follow-up, Nt-pro-BNP was lower in the exercise groups compared to UC. At 2-years there was a drop in VO_2peak_ for patients with high cTnT and Nt-pro-BNP. Fewer patients in the RT-HIIT group fulfilled biomarker risk criteria compared to UC (OR 0.200; 95% CI = 0.055–0.734).

**Conclusions:**

In this cohort, high-intensity exercise was associated with lower levels of NT-proBNP 1-year post-baseline, but not with cTnT directly after treatment completion. This may, together with the preserved VO_2peak_ in patients with low levels of biomarkers, indicate a long-term cardioprotective effect of exercise.

**Trial registration:**

Clinicaltrials.govNCT02522260, Registered 13th of august 2015 – Retrospectively Registered

**Graphical abstract:**

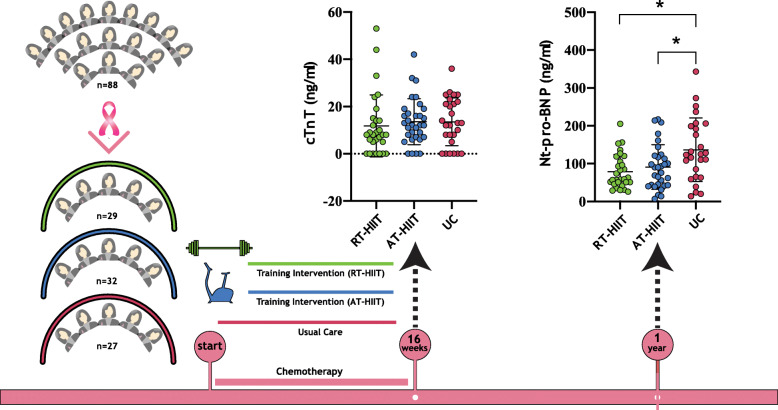

**Supplementary Information:**

The online version contains supplementary material available at 10.1186/s40959-021-00091-1.

## Introduction

Cardiovascular disease is the second leading cause of morbidity and mortality among breast cancer survivors after recurrence [1]. The risk of cardiovascular mortality is twice as high in this population compared to age matched individuals [2–4].

The main culprit for cardiac toxicity in the wake of chemotherapy are anthracyclines [3, 5, 6]. Anthracyclines are highly effective in the treatment of breast cancer, however, the use of these agents have been limited by cumulative, progressive and dose-dependent cardiotoxicity [7, 8], presenting with worse prognosis than idiopathic cardiomyopathy [9]. Anthracyclines are often used in combination with other potentially cardiotoxic agents, such as trastuzumab, and/or thoracic radiation therapy (RT). These combinations improve breast cancer specific outcomes but may further compromise cardiac function [10, 11].

Plasma cardiac Troponins (cTnT), a biochemical marker of myocardial injury, has been shown to increase acutely in response to anthracycline therapy [12–15]. In pediatric patients and patients with breast cancer treated with high-dose chemotherapy, cTnT release during chemotherapy was predictive for decreases in left ventricular ejection fraction (LVEF) and other echocardiographic parameters, as well as clinical cardiac events after treatment [12–14]. NT-pro-BNP, a natriuretic peptide used in diagnosis and assessment of severity of heart failure, has also been investigated in patients treated with anthracyclines, and persistent Nt-pro-BNP elevations at 1-year post treatment has been associated with decreased LVEF [16]. Identifying preventive measures to reduce therapy-induced cardiotoxicity is important to ensure the long-term health of patients who have received curative treatment for breast cancer [17, 18].

The OptiTrain study, and others, have shown the benefits of exercise in reducing treatment side effects such as fatigue, reduced fitness and muscle strength for patients with breast cancer [19–24]. Implications of exercise on the cardiotoxicity caused by cancer therapies is currently under investigation, and concrete data from randomized trials are warranted to further this field [25]. Two studies have addressed the effects of exercise during anthracycline treatment on cardiac biomarkers post intervention [26, 27], showing no effects of exercise on Troponin I release or reduction in resting LVEF [26, 27]. To the best of our knowledge, no study has addressed long term effects on cardiotoxicity from participating in an exercise intervention during therapy.

The OptiTrain randomized control trial (RCT) was designed to compare the effects of resistance and high-intensity interval training (RT-HIIT), moderate-intensity aerobic and high-intensity interval training (AT–HIIT), to usual care (UC) during adjuvant chemotherapy for early breast cancer [2, 17, 28, 29]. In this post-hoc exploratory analysis of the OptiTrain trial, the effects of exercise on acute and chronic therapy-induced cardiotoxicity were monitored by assessing cardiorespiratory fitness and serum levels of cTnT and Nt-proBNP over the intervention and into survivorship.

## Materials & methods

### Ethical approval

All procedures performed were in accordance with the Helsinki Declaration and ethical standards of the institutional and national research committee (regional ethical review board in Stockholm, Sweden. Dnr 2012/1347–31/1, 2012/1347–31/2, 2013/7632–32, 2014/408–32, 2016/57–32, 2018/446–32, 2019/013–10).

### Study design

Between March 2013 and July 2016, two hundred and forty patients from the Karolinska University Hospital and Södersjukhuset, Stockholm, Sweden were eligible for and accepted participation in the OptiTrain study (NCT02522260) [30]. Inclusion criteria were Swedish-speaking women, 18–70 years old diagnosed with breast cancer stage I–IIIa and scheduled to undergo chemotherapy consisting of anthracyclines, taxanes, or a combination of the two. Exclusion criteria for the trial were pre-existing cardiac pathologies (assessed by routine electrocardiograms and a questionnaire), major psychiatric disorders, or other concurrent malignant diseases. All participants gave written informed consent prior to enrolment. The participants were randomly allocated to resistance and high-intensity interval training (RT–HIIT), moderate-intensity aerobic and high-intensity aerobic interval training (AT–HIIT), or usual care (UC). The original OptiTrain RCT protocol and intention to treat analyses of the 16-week, 1 and 2-year-follow-up have been published [19, 30–34]. In the current study we used a per protocol analysis. Inclusion criteria were attendance to at least 16 supervised training sessions (per protocol analyses) and to have provided blood samples at baseline, at 16-weeks, and at the 1-year- follow-up.

### Intervention

The RT–HIIT and AT–HIIT groups undertook supervised exercise sessions at the in-hospital exercise clinic, twice weekly for 16-weeks as previously described [19]. In short, the RT-HIIT group performed resistance training consisting of 8–12 repetitions at 75–80% of 1RM targeting the major muscle groups, followed by 3 × 3 min bouts of aerobic high intensity interval training (HIIT) on a cycle ergometer. The AT-HIIT group started each session with 20 min of moderate intensity continuous aerobic exercise followed by the same HIIT regimen as RT-HIIT. The UC group was given written information at the initiation of the intervention period about exercise recommendations for patients with cancer, according to 2010 American College of Sports Medicine exercise guidelines for cancer survivors [35].

### Outcome measures

Outcomes were assessed at baseline and after the intervention period (16-weeks), and at one, two and 5 years (5 year-assessments are currently ongoing and not included in this study) post-baseline. Objectively measured sedentary behaviour and physical activity were assessed by accelerometer (model GT3X ActiGraph) at baseline and estimated VO_2peak_, as a proxy for cardiorespiratory fitness (CRF), was assessed at baseline and at all follow up timepoints using the Åstrand Rhyming submaximal cycle test as previously described [31]. VO_2peak_ is presented in the study expressed as litres per min (l/min) to avoid the impact of body mass fluctuations. The submaximal exercise test for assessment of CRF has been validated in patients with breast cancer undergoing chemotherapy [36]. Blood samples were collected at baseline, at the 16-week timepoint and at the 1-year follow-up.

### Biomarkers

Cardiac Troponin T was selected based on its sensitivity in detecting acute cardiac damage, and is validated as a biomarker for anthracycline-related cardiotoxicity [8]. NT-pro-BNP is a biomarker for long-term cardiac remodelling, secreted by cardiomyocytes in response to cardiac wall stress [37]. Plasma cTnT and NT-pro-BNP were analysed by the clinical laboratory at Karolinska University Hospital using electrochemiluminescense immunoassay, ECLIA. The high-sensitivity cardiac troponin T (hs-cTnT) assay (Elecsys hs-cTnT assay Roche Diagnostics, Mannheim, Germany) has a limit of detection (LoD) of 5 ng/l, a 99th percentile value of 14 ng/l and a coefficient of variance (CV) of 10% at 13 ng/l.

### Biomarker dependent stratification to assess risk of decreased cardiovascular function

Values of cTnT after completion of chemotherapy and Nt-pro-BNP at the 1-year follow up were used to identify patients at risk for decreased cardiovascular function. The biomarker criteria were set to cTnT > 10 ng/ml after the treatment and Nt-pro-BNP > 100 ng/ml at the 1-year follow up.

### Statistical analysis

Data were analysed using the IBM SPSS Statistics V. 25.0 statistical package. Baseline medical and demographic characteristics were summarized using descriptive statistics. One-way ANOVA and exact χ2 (chi2) tests were used to evaluate if differences existed between groups at baseline. For between-groups analyses of the biomarkers, analysis of covariance (ANCOVA), adjusted for baseline moderate to vigorous physical activity level (MVPA), was used. Between-groups differences in VO_2peak_ were adjusted for baseline values and baseline MVPA. To determine within group change in circulation of biomarkers repeated measures ANOVA was used. Spearman’s coefficient of correlation (r) was used to evaluate the association between age and the release of biomarkers as well as the association between cTnT and Nt-pro-BNP. The distribution of 1-year Nt-pro-BNP levels divided into 16-week cTnT intervals was analysed with one-way ANOVA. Odds ratios were computed as the incidence in the intervention group compared to the incidence in the control group. All tests were 2-tailed and *p* < 0.05 was considered significant.

## Results

### Inclusion criteria

**Fig. 1:**
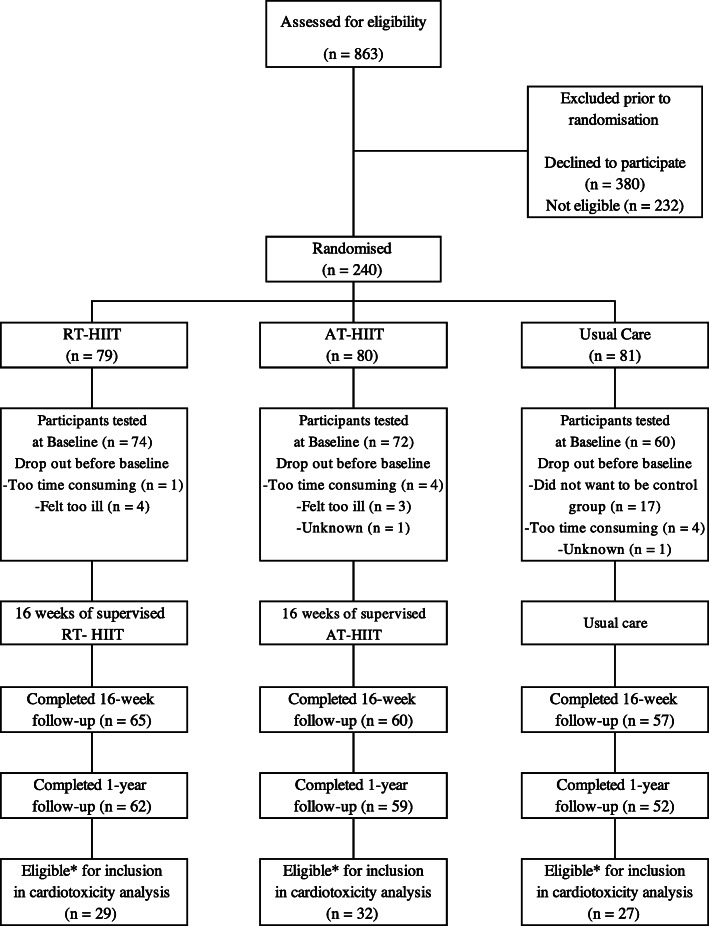
CONSORT diagram. RT–HIIT resistance and high-intensity interval training, AT–HIIT moderate-intensity aerobic and high-intensity interval training, UC usual care. *Eligibility for inclusion in cardiotoxicity analysis was determined by completion of at least 16 out of 26 supervised training sessions and blood samples from baseline, after chemotherapy, and at the 1-year follow up

In total, 240 women were recruited to the OptiTrain study [30]. These post-hoc analyses were performed using a per-protocol analysis approach based on the following requirements: A) attendance at a minimum of 16 (60%) supervised training sessions for those in the exercise groups and B) having contributed blood samples at baseline, at 16-weeks, and at the 1-year follow up. The number of participants that met the inclusion requirements were *n* = 29 from RT-HIIT, *n* = 32 from AT-HIIT, and *n* = 27 from the control group (Fig. 1).

### Baseline characteristics

The groups were balanced in terms of baseline demographics (Table 1). There was, however, a baseline difference in moderate to vigorous physical activity (MVPA), with the RT-HIIT group recording more active minutes per day (*p* < 0.001) compared to the AT-HIIT and UC group at baseline. Four patients had a pre-existing hypertension diagnosis at baseline. There were no significant differences between groups at baseline with regards to tumor HER-2 positivity or anthracycline and radiotherapy treatment.

**Table 1:** RT–HIIT resistance and high-intensity interval training, AT–HIIT moderate-intensity aerobic and high-intensity interval training, UC usual care, SD standard deviation, BMI body mass index, HER human epidermal growth factor receptor positivity, ER estrogen receptor positivity, MVPA objectively measured moderate- to vigorous-intensity physical activity, SED objectively measured sedentary behaviour. *One-way ANOVA, † Exact χ2 test, ** denotes *p* < 0.01

Participant characteristics at baseline				
	RT-HIIT *n* = 29	AT-HIIT *n* = 32	Usual care *n* = 27	
	**Mean ± SD**	**Mean ± SD**	**Mean ± SD**	*p*-value*
**Age**	53.5 ± 10.2	53.7 ± 7.9	55.9 ± 7.5	0.52
**Body mass (kg)**	65.8 ± 9.4	67.0 ± 10.3	69.7 ± 12.5	0.40
**Height (cm)**	165.5 ± 6.1	166.6 ± 6.2	165.6 ± 6.3	0.75
**BMI**	24.0 ± 2.9	24.2 ± 3.3	25.4 ± 4.4	0.26
**SED (min/day)**	551.0 ± 102.3	534.2 ± 86.4	567.7 ± 68.03	0.34
**MVPA (min/day)**	97.7 ± 35.1	81.4 ± 24.7	70.0 ± 31.7	0.01**
	**%**	**%**	**%**	***p*-value†**
**Married or partnered**	69.0	62.5	63.0	0.78
**University level education**	69.0	68.8	59.3	0.55
**Postmenopausal**	51.7	59.4	74.0	0.22
**Smoker**	3.4	3.1	3.7	0.99
**Radiotherapy**	86.2	87.5	85.2	0.97
**Anthracycline based chemotherapy**	93.1	93.8	100	0.39
**Tumor receptor status**				0.76
**Triple negative**	7	15.6	14.8	
**HER2+, ER±**	27.5	31.3	22.2	
**HER2−, ER+**	65.5	53.1	59.3	
**HER2−, ER−**	0	0	3.7	

### Plasma cTnT levels

**Fig. 2:**
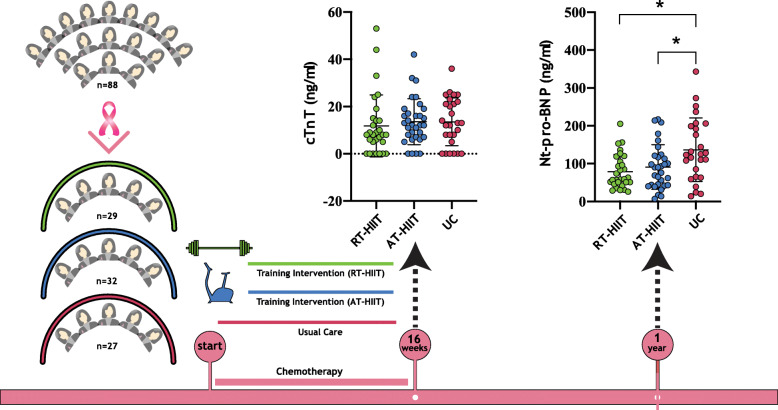
Plasma biomarkers. Left panel, Plasma levels of cTnT post treatment (16-weeks) per training allocation. RT–HIIT resistance and high-intensity interval training, AT– HIIT moderate-intensity aerobic and high-intensity interval training, UC usual care. Right panel, Plasma Nt-pro-BNP levels between the groups at the 1-year follow up. ANCOVA. Data is presented as scatterplots and mean ± SD, * denotes *p* < 0.05

Baseline median cTnT was below detection limit in all groups. After completion of chemotherapy (16-week time point), the median/mean level of cTnT was significantly higher in all groups compared to baseline, with no difference between the groups, suggesting a treatment-induced increase in cTnT (Fig. 2 and Supplementary Fig. 1A).

### Plasma Nt-pro-BNP levels

There were no significant differences in median/mean levels of Nt-pro-BNP between the three groups at baseline or after completion of chemotherapy (16-week time point). The median/mean level of Nt-pro-BNP at 1 year follow up was higher in the UC group compared to RT-HIIT and AT-HIIT (*p* = 0,036) (Fig. 2 and supplementary Fig. 1B).

### Cardiorespiratory fitness

At 16-weeks there was a difference in median/mean VO_2peak_ between the groups. RT-HIIT and AT-HIIT maintained cardiorespiratory fitness, while there was a decline in the UC group (*p* = 0.013), in line with the findings from the intention to treat analyses previously reported [19]. Similar to the previously reported intention to treat analyses of this OptiTrain study, there was no between-groups difference in VO_2peak_ at the 1- and 2-year follow up time points (supplementary Fig. 2A-C) [31, 32].

### Correlations between levels of cTnT and Nt-pro-BNP

**Fig. 3:**
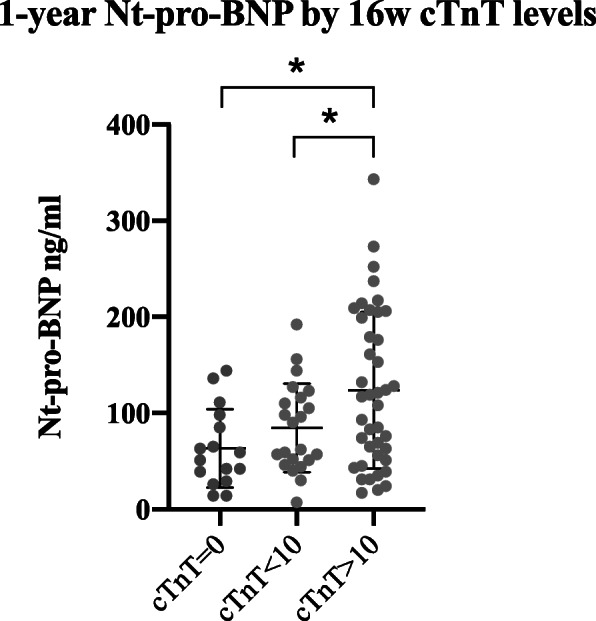
Levels of Nt-pro-BNP divided into groups of no TnT release, TnT < 10, and TnT > 10. One-way ANOVA Data is presented as scatterplots and mean ± SD, * denotes *p* < 0.05

There was a statistically significant correlation between levels of cTnT at the 16-week time point and of Nt-pro-BNP at the 1-year follow up time point (*p* = 0.0047, *r* = 0.30). Further dividing the 16-week cTnT release into three categories; undetectable cTnT levels, cTnT > 0 ng/ml but < 10 ng/ml, and cTnT> 10 ng/ml, showed higher 1-year levels of Nt-pro-BNP in the group with the highest cTnT at 16-weeks (*p* = 0.0015) (Fig. 3).

### Risk stratification

The biomarker criteria for risk of decreased cardiac function were 1) cTnT > 10 ng/ml post-intervention and 2) Nt-pro-BNP > 100 ng/ml at the 1-year follow up time point. The cut-off for cTnT, 10 ng/ml, was also put forward in previous studies as having predictive value of cardiac dysfunction associated with anthracycline therapy [8, 13, 15]. The Nt-pro-BNP cut-off was set to 100 ng/ml, based on the across observations median level at the 1-year follow-up time point being close to 100 ng/mL, a value previously used in primary care settings as a cut-off for referrals from general physicians to cardiologists [28, 38].

**Fig. 4:**
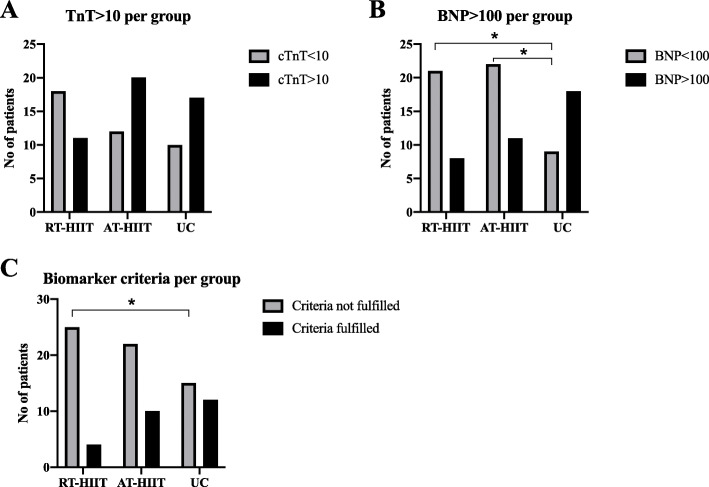
Number of patients with release of cTnT of > 10 (**a**) & Nt-pro-BNP > 100 (**b**) by training allocation. RT–HIIT resistance and high-intensity interval training, AT–HIIT moderate-intensity aerobic and high-intensity interval training, UC usual care. **c** Number of patients fulfilling biomarker criteria by training allocation. RT–HIIT resistance and high-intensity interval training, AT–HIIT moderate-intensity aerobic and high-intensity interval training, UC usual care. χ2 between group analysis. * denotes *p* < 0.05

There were more patients with 1-year Nt-pro-BNP > 100 in the UC group than in the training groups (*p* = 0.006) (Fig. 4b). There was also fewer patients fulfilling the biomarker criteria for risk of decreased cardiac function from the RT-HIIT group compared to AT-HIIT and UC (*p* = 0.04) (Fig. 4c). The odds ratio for a patient in the usual care group to fall in this category compared to a patient allocated to RT-HIIT was 0.200 (95% CI = 0.055–0.734). The corresponding odds ratio for a patient in UC group compared to AT-HIIT was 0.568(95% CI = 0.196–1.649).

**Fig. 5:**
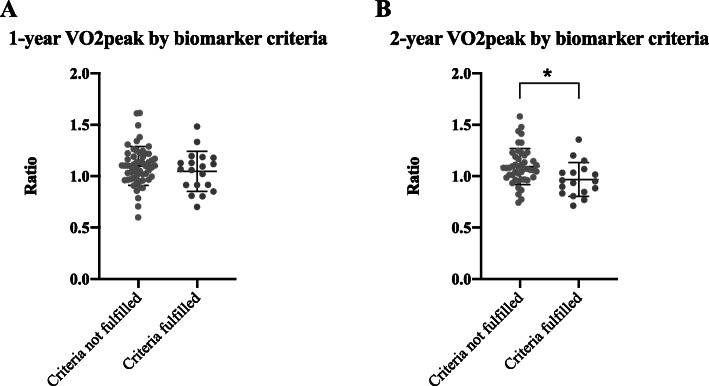
Change in VO_2peak_ (expressed as ratio of baseline) between baseline and 1-year (**a**) and 2-year (**b**) follow-up respectively, separated by patients fulfilling biomarker criteria and patients not fulfilling the biomarker criteria. Data is presented as scatterplots and mean ± SD, ANCOVA * denotes *p* < 0.05

Of note, patients fulfilling the biomarker criteria showed a lower VO_2peak_ ratio, and a decrease in cardiorespiratory fitness, from baseline to the 2-year follow up, which was significantly different from the patients not fulfilling the criteria (*p* = 0.022) (Fig. 5b). The 2-year follow up from baseline VO_2peak_ ratio for the patients fulfilling the risk criteria was 0.97 (±0.17) meaning an on-average decline by 3%, while the patients not fulfilling the risk criteria had a 2-year to baseline ratio of 1.09 (±0.18), representing an increase in VO2_peak_ of 9%. These findings suggest that these biomarker criteria are of relevance to cardiorespiratory fitness, and thereby cardiovascular function, in this patient population.

## Discussion

In this post-hoc analysis of participants of the OptiTrain trial, we found that median levels of cTnT was increased after chemotherapy completion in both exercise groups and in patients receiving usual care. At the 1-year follow up, plasma Nt-pro-BNP was significantly lower in the exercise groups compared to the usual care group. At 2-years there was a significant drop in VO_2peak_ amongst patients with a more pronounced release of cTnT and NT-proBNP, indicating that the early change in biomarkers point to a patient group with compromised cardiovascular function.

Cardiorespiratory fitness, measured as VO_2peak_, is highly predictive of overall and cardiovascular specific mortality in women, both in the healthy population and in a breast cancer setting [26, 39, 40]. VO_2peak_ has been shown to decline between 5 and 26% during exposure to anthracycline regimens, and many patients do not recover to their baseline cardiorespiratory fitness after treatment completion [41, 42]. Exercise is known to preserve cardiorespiratory fitness in this patient population, indicating that exercise prescription before, during, and/or after treatment could provide cardioprotective benefits [19, 22, 26, 27]. Whether exercise can reduce the acute cardiotoxicity of anthracycline treatment or mitigate pathological remodelling and thereby reduce the risk of heart failure in this population is currently unknown.

Investigating biomarkers of acute cardiomyocyte damage and long-term ventricular remodelling in the OptiTrain study showed a statistically significant release of cTnT in most patients after chemotherapy. Out of the 88 patients included, 48 had levels of 10 ng/ml or more after completing chemotherapy treatment, the level usually defined as being associated to subclinical treatment-related cardiotoxicity [8, 13, 15]. The time point for the blood sample referred to as post treatment (16-weeks), from which this biomarker was analyzed, ranged from 18 to 24 days following the patient’s last cycle of chemotherapy [30]. Troponin is released in response to acute myocardial damage and the serum concentration increases within 3 to 6 h and thereafter slowly declines over the course of 7 to 10 days [43]. In our analysis, the cTnT release did not vary significantly between the three different groups, suggesting that physical exercise does not ameliorate acute cardiomyocyte damage related to chemotherapy treatment. These findings are in line with previous exercise trials by Campbell et al. and Howden et al. [26, 27] that showed no difference in levels of troponin between exercise groups and usual care. Of note, the large proportion of patients in this trial with elevated cTnT levels (80%), are in contrast to previous studies, where detectable serum levels of cTnT were found in only 18–30% of the patients, with very few above 10 ng/ml [8, 13, 15]. The increased detection may be a result of differences in the chemotherapy regimens or the sensitivity of the method for analysis of cTnT. In the current study the highly sensitive ECLIA method was used for detection of cTnT.

In line with the current findings, earlier studies have reported no change in Nt-pro BNP when measured immediately after chemotherapy treatment completion [26, 27] In the OptiTrain study, the difference in Nt-pro-BNP was only found at the 1-year time point, and to date, this is the first report of long-term follow up on biomarkers of cardiotoxicity from an exercise intervention. The significant difference between the intervention groups and the UC group regarding Nt-pro-BNP at the 1-year follow up is novel and possibly of clinical importance. The finding might be interpreted as a difference in the magnitude of structural cardiac remodelling between the patients in the exercise groups and the UC group.

A release in cTnT at 16-weeks was associated with higher Nt-pro-BNP levels at a later stage (1-year follow up). Importantly, when defining a group with risk of decreased cardiac function, fulfilling the criteria of cTnT> 10 ng/ml post treatment and Nt-pro-BNP > 100 ng/ml after 1 year, a decline in VO_2peak_ by 3% was found, compared to the 9% increase in the rest of the study sample. Fulfilling the biomarker criteria was much more probable for patients who were allocated to UC than the participants in the RT-HIIT group.

### Strengths and limitations

The main strengths of the OptiTrain trial are the RCT design, the long term follow up, and the relatively large sample size. Available studies looking at effects of exercise on cardiotoxicity rely on substantially smaller numbers of patients and only include data to the end of the treatment or intervention. To follow patients from baseline to a 2-year follow-up provides unique opportunities to look at both the short- and long-term effects of an exercise intervention in a defined population. Another strength is that two types of supervised exercise regimens, RT-HIIT and AT-HIIT, were trialled in parallel during the chemotherapy treatment. All the exercise sessions were standardized, supervised and performed in the hospital gym allowing for a controlled environment. The biomarkers reported were analysed in a standardized manner at the clinical laboratory at Karolinska University hospital, where blood samples are routinely handled, making that data comparable to results from other clinical studies.

A limitation of the interpretation of the current data is the significant difference between groups in MVPA at baseline when using the per protocol approach. As discussed previously, the difference between the groups regarding baseline moderate to vigorous physical activity level (MVPA) should be taken into consideration, and although MVPA was included as a covariate in the statistical analyses, the results of this study must be interpreted with caution. Another limitation is that VO_2peak_ was estimated from a submaximal test. However, in a situation where, maximal exercise testing is not practical or desirable from a patient safety perspective, submaximal exercise testing provides an alternative way of estimating VO_2peak_. The Åstrand-Rhyming prediction model has been validated as a submaximal exercise test for determining cardiorespiratory fitness in this population [36], although its sensitivity to change has not been determined.

### Clinical implications

The results of this study indicate that exercise during chemotherapy is not associated with any significant risks for the patient with regards to biomarkers of acute and long-term cardiotoxicity. Given that cardiovascular disease exceeds breast cancer as the leading cause of death 7 years after their breast cancer diagnosis [44], strategies to maintain cardiorespiratory fitness and function cannot be emphasized enough. Our results indicate that exercise, and RT-HIIT in particular, may provide a means to mitigate myocardial damage in the long term.

### Future studies

The mechanisms by which exercise training confers positive benefits in breast cancer survivors remain to be determined. Future studies designed to assess cardiotoxicity and cardio-protection in this population should consider including extensive baseline and follow-up testing of cardiac function and additional follow-up time-points to better capture peak levels of biomarkers. Building on the indication that RT-HIIT has cardio protective effects, further investigation is merited. To investigate the effects of exercise on structural differences regarding resting heart function, the use of echocardiograms at baseline and follow-up could add relevant information about changes in cardiac wall thickness, ejection fraction, global longitudinal strain, tricuspid insufficiency, and maximal velocities. To further develop and integrate exercise interventions in clinical oncology, it is important to conduct randomized clinical trials and assess the effects of exercise to differentiate cardioprotective effects of exercise during chemotherapy from mitigation of cardiovascular risk factors.

## Conclusions

In this cohort, high-intensity exercise was associated with lower levels of NT-proBNP 1-year post-baseline, but not with acute cTnT rises directly after treatment completion. This may, together with the preserved VO_2peak_ in patients with low levels of biomarkers, indicate a long-term cardioprotective effect of exercise in patients with early breast cancer.

## Supplementary Information

**Additional file 1: Supplementary Fig. 1**. Median cTnT for all timepoints by training allocation. γ denotes *p* < 0.05 in all groups (RM-ANOVA). * denotes *p* < 0.05 between groups (ANCOVA).

**Additional file 2: Supplementary Fig. 2**. VO_2peak_ for all timepoints by training allocation. Data is presented as scatterplots and mean ± SD, * denotes *p* < 0.05 (ANCOVA).

## Data Availability

The datasets used and/or analysed during the current study are available from the corresponding author on reasonable request.
