# Supplementary figures and images for: High intensity exercise during breast cancer chemotherapy - effects on long-term myocardial damage and physical capacity - data from the OptiTrain RCT
Source: Cardiooncology. 2021 Feb 15;7:7. doi: 10.1186/s40959-021-00091-1 (PMC7883413; doi:10.1186/s40959-021-00091-1)

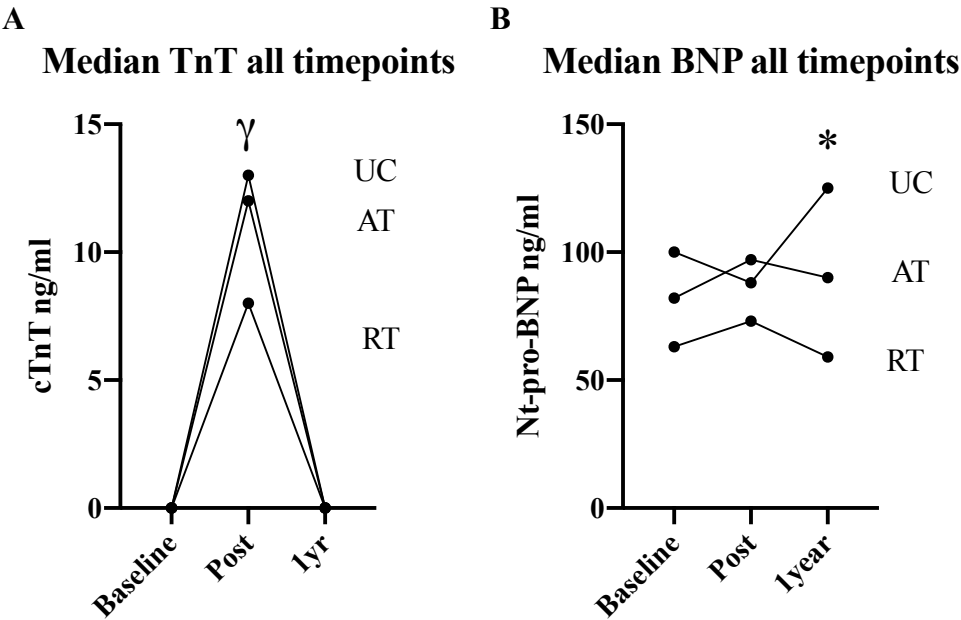

Supplement: Supplementary file 1 — Additional file 1: Supplementary Fig. 1. Median cTnT for all timepoints by training allocation. γ denotes p < 0.05 in all groups (RM-ANOVA). * denotes p < 0.05 between groups (ANCOVA). [file 40959_2021_91_MOESM1_ESM.pdf]

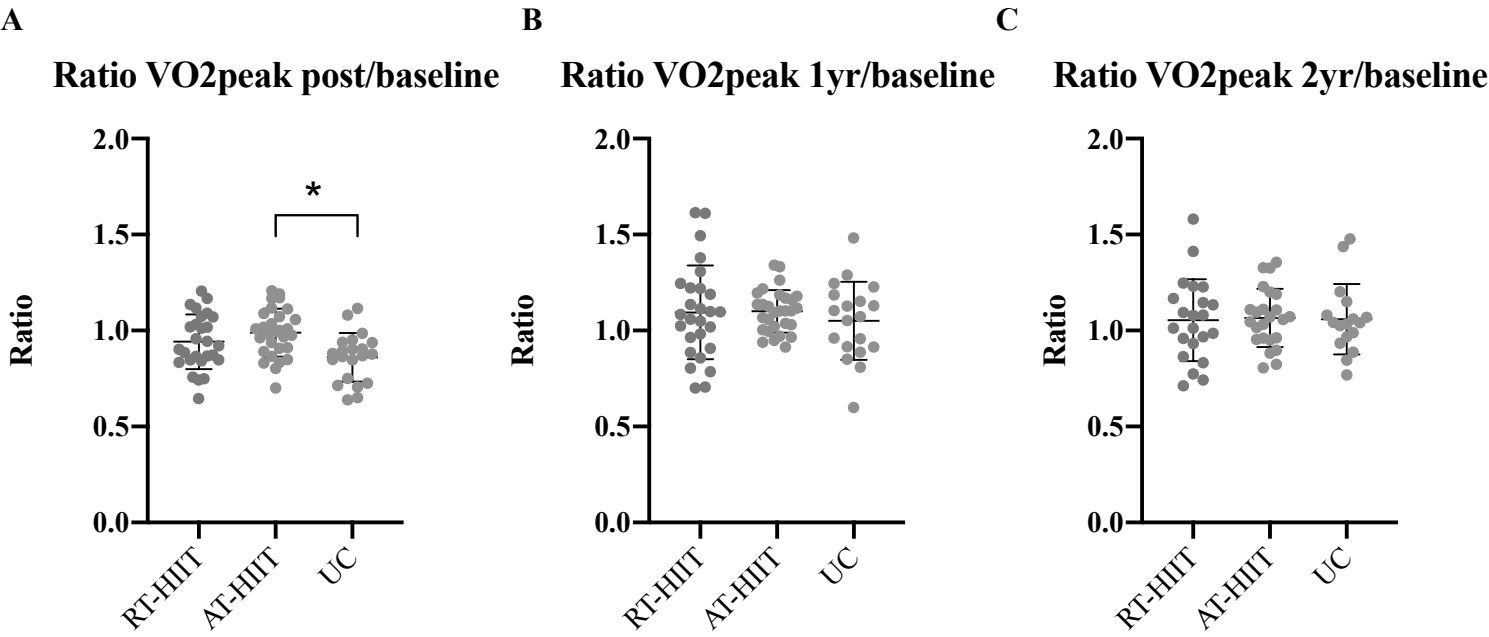

Supplement: Supplementary file 2 — Additional file 2: Supplementary Fig. 2. VO2peak for all timepoints by training allocation. Data is presented as scatterplots and mean ± SD, * denotes p < 0.05 (ANCOVA). [file 40959_2021_91_MOESM2_ESM.pdf]
